# Blockchain-Based Digital Twins Collaboration for Smart Pandemic Alerting: Decentralized COVID-19 Pandemic Alerting Use Case

**DOI:** 10.1155/2022/7786441

**Published:** 2022-01-13

**Authors:** Radhya Sahal, Saeed H. Alsamhi, Kenneth N. Brown, Donna O'Shea, Bader Alouffi

**Affiliations:** ^1^SMART 4.0 Fellow, School of Computer Science and Information Technology, University College Cork, Cork, Ireland; ^2^Faculty of Computer Science and Engineering, Hodeidah University, Al Hudaydah, Yemen; ^3^SMART 4.0 Fellow, Technological University of the Shannon, Midlands Midwest, Athlone, Ireland; ^4^Faculty of Engineering, IBB University, Ibb, Yemen; ^5^School of Computer Science and Information Technology, University College Cork, Cork, Ireland; ^6^Department of Computer Science, Munster Technological University, Cork, Ireland; ^7^Department of Information Technology, College of Computers and Information Technology, Taif University, P. O. Box 11099, Taif 21944, Saudi Arabia

## Abstract

Emerging technologies such as digital twins, blockchain, Internet of Things (IoT), and Artificial Intelligence (AI) play a vital role in driving the industrial revolution in all domains, including the healthcare sector. As a result of COVID-19 pandemic outbreak, there is a significant need for medical cyber-physical systems to adopt these emerging technologies to combat COVID-19 paramedic crisis. Also, acquiring secure real-time data exchange and analysis across multiple participants is essential to support the efforts against COVID-19. Therefore, we have introduced a blockchain-based collaborative digital twins framework for decentralized epidemic alerting to combat COVID-19 and any future pandemics. The framework has been proposed to bring together the existing advanced technologies (i.e., blockchain, digital twins, and AI) and then provide a solution to decentralize epidemic alerting to combat COVID-19 outbreaks. Also, we have described how the conceptual framework can be applied in the decentralized COVID-19 pandemic alerting use case.

## 1. Introduction

Healthcare 4.0 is a crucial enabler for transforming care to be autonomous by building a learning healthcare system to empower data-driven-based decision. It uses the Industrial Internet of Things (IIoT), digital twins, Machine Learning (ML), and big data technologies [1, 2]. Digital twins represent a virtual replica of physical things, including devices attached to humans and machines. Combining the digital twins technologies with blockchain is making the right way to build MCPS based on secure interconnections of several devices and systems in the medical field [3, 4]. Furthermore, the effective interconnections among the devices need a secure decentralized collaborative system to provide timely notifications to the decision-makers in MCPSs [5, 6]. Furthermore, COVID-19 pandemic time confirms the need for using the capability of combining the advanced technologies (i.e., blockchain, digital twins, and AI) to enable and enhance remote work during the lockdown and to avoid worse coronavirus outbreaks [7].

The different participants are represented by multiple digital twins, which could be deployed within the MCPS. These deployed digital twins provide the MCPS for the dynamic updates of the participants, including their status and activities [8]. The deployed digital twins within MCPSs are intelligently collaborating and interacting to perform real-time predictions to avoid any potential risks of coronavirus spread. They communicate effectively to share their data and learn using shared knowledge about the medical historical logs. The predicted potential risks contribute to the MCPS's decision-making process to combat pandemic outbreaks through announcing quarantine, lockdown, and restrictions. However, the digital twin paradigm is still at an early stage, and many challenges still exist in adopting digital twins collaboration in the decentralized systems, including the following:

- *Interoperability.* We have to define the models and strategies of sharing policies (i.e., internal and external data) through identifying the digital twins' data schema and the collaboration requirements.
- *Authentication*. In some scenarios in decentralized systems, the deployed digital twins are owned by independent entities that want to collaborate. Therefore, the decentralized digital system needs secure and efficient technology to acquire secure real-time data exchange and analysis across multiple participants.
- *Distributed Machine Learning*. A large-scale input data size from multiple participants needs to be analyzed to provide accurate predictions about the potential risks within the decentralized system.
- *Decentralized Decision-Making*. Centralizing suffers from single failure data, while decentralization suffers from lacking global data. Therefore, a decision-making consensus is required.
- *Scalability and Robustness*. A system needs to accommodate a large number of digital twins which represent multiple participants, e.g., objects, devices, machines, nodes, people, workstations, and so on, within decentralized systems. Also, the decentralized system needs to deal with multiple deployed digital twins and simultaneously maintain the robustness at a required level, especially with the hacked nodes and malfunctioning cases.

Consequently, most of research studies have proposed adopting blockchain with digital twins to guarantee transparency, decentralized data storage, data sharing, peer-to-peer communication, secure traceability, and scalability [9]. Suhail et al. [10] have discussed the research trends, issues, and future challenges. They have presented a comprehensive review of the state-of-the-art research results for blockchain-based digital twins. Furthermore, using multiple blockchain-based digital twins can collaborate in a hierarchical and granular manner by using shared knowledge to manage and trace the product assembly data [11]. Moreover, a smart contract is used to execute some actions automatically to efficiently increase data sharing and provide higher security [12]. It provides trusted data provenance, audit, traceability, and tracking transactions initiated by participants involved in the creation of digital twins [6].

Regarding COVID-19 combating, Alsamhi and Lee [13] discussed the integration of blockchain for multirobots collaboration to combat COVID-19 in different scenarios. Furthermore, blockchain helps multidrones to collaborate in a decentralized fashion of the peer-to-peer network to fight COVID-19 [7]. However, the existing research lacks solutions for collaborative digital twins based on data analytics because it focuses mainly on blockchain adoption. Therefore, there are still many challenges requiring further investigation to identify the potential risks in decentralized systems using the intelligence of the digital twin-driven data.

### 1.1. Contributions

This decentralized pandemic alerting framework is proposed to provide more intelligent and collaborative solutions for digital twins based on blockchain. The proposed decentralized alerting framework is considered one type of decentralized apps (dApp) that could integrate blockchain and digital twins to combat COVID-19 and any future pandemics. The proposed framework could be developed and implemented in the MCPS, and it utilizes the blockchain to guarantee transparency, decentralized data storage, data sharing, peer-to-peer communication, secure traceability, and scalability for smart healthcare.

**Figure 1:**
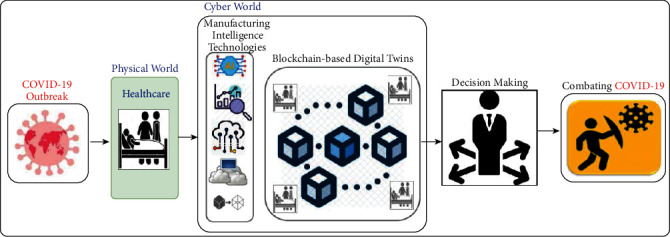
The high level of the blockchain-based collaborative digital twins for MCPS to combat COVID-19.

Therefore, the main purpose of this work is to develop a framework for blockchain-based collaborative digital twins to introduce decentralized COVID-19 pandemic alerting. In particular, the proposed framework targets providing a smart decentralized pandemic alerting framework for predicting the potential risks using the intelligence of sharing digital twin-driven data.  Figure 1 depicts the high level of the blockchain-based collaborative digital twins for the MCPS to combat COVID-19. As it can be noticed, the physical world is the people in the healthcare system; these people can be patients, doctors, nurses, and so on. On the other hand, the cyber world introduces advanced technologies such as blockchain, digital twins, AI, big data, edge computing, and cloud computing. These advanced technologies are working together to provide insightful information to decision-makers. According to the MCPS, the decision-making participants could be hospitals, health organizations, and governments. The data-driven decision is made based on the potential risks of COVID-19 outbreaks, which leads to announcing quarantine, lockdown, or some restrictions to combat pandemic outbreaks.

Our main contributions in this research paper can be summarized as follows:

- We discuss the combination of blockchain, digital twins, and data analysis technologies in terms of the benefit of combining these advanced technologies, blockchain-based decentralization, and the concept of digital twins collaboration.
- We propose the conceptual framework of blockchain-based digital twins collaboration for pandemic alerting. The proposed framework utilizes the data-driven digital twins collaboration with the help of blockchain technology to combat pandemics such as COVID-19. Four layers are introduced to equip the conceptual framework of blockchain-based digital twins collaboration with operational data intelligence, namely, the physical layer, blockchain-based digital twins layer, data analytics layer, and decision-making layer. In addition, the physical layer contains human and nonhuman participants.
- We describe how the conceptual framework can be applied in a decentralized COVID-19 pandemic alerting use case. In particular, we address the decentralized pandemic alerting to combat COVID-19.

### 1.2. Paper Organization

The remainder of this paper is organized as follows: combination of blockchain, digital twins, and data analysis is introduced in Section 2. This section introduces the benefits of combining the advanced technologies and digital twins collaboration concept. The proposed conceptual framework for blockchain-based collaborative digital twins for pandemic alerting is presented in Section 3. The use case of decentralized COVID-19 pandemic alerting is presented in Section 4. The related work which contains a comparison with other existing solutions of blockchain and digital twins is introduced in Section 5. The discussion and future directions are presented in Section 6. Finally, conclusions are presented in Section 7.

## 2. Combination of Blockchain, Digital Twins, and Data Analysis Technologies

In this section, we discuss the decentralization for blockchain, the combination of blockchain, digital twins, and data analysis technologies in terms of the benefits of combining these advanced technologies, and the concept of digital twins collaboration.

### 2.1. Understanding Blockchain-Based Decentralization

Decentralization is a concept of transferring decision-making and control from centralized into distributed networks. While decentralized networks are often used in blockchain technology, a blockchain application cannot be classified as decentralized or not. Perhaps, decentralization should be extended to all aspects of a blockchain program on a sliding scale. More noticeable and pleasant support may be achieved by decentralizing and accessing assets in an application. The use of blockchain in Industry 4.0 can improve the notion of digital twins through assuring transparency, data immutability, decentralized data storage, and peer-to-peer communication. Furthermore, a lot of research work has addressed the decentralization feature of blockchain for several applications [9, 14, 15].

All nodes in a centralized network are connected to a single authority. However, the decentralized network does not have a single authoritative server that governs all nodes; instead, each node has its entity. As a result, decentralization suffers from a lack of global data, while centralization suffers from single failure data [14]. Thus, decision-making is necessary for digital twin collaboration. The consensus-based distributed decision-making process is insightful and produces efficient and dependable collaborative solutions in terms of substance.

The Directed Acyclic Graph (DAG)-structured distributed ledger technology (DLT) solution will be considered as part of its consensus mechanism, which will allow for a completely decentralized manufacturing environment [16, 17]. Additionally, DLT can assure that all participants (i.e., deployed digital twins) have the same information, allowing for the prediction that requires a worldwide agreement on the object of interest (e.g., fault diagnosis) [5]. This agreement will be achieved via the consensus method, which uses the local interaction of the deployed digital twins within a manufacturing node and then the ledger-based running database to create a worldwide agreement [18].

### 2.2. Benefits of Combining Blockchain, Digital Twins, and Data Analysis Technologies

The key benefit of using digital twins with blockchain technology is storing information from the digital twins and interaction between digital twins and blockchain. This allows digital twins to enter into smart contracts with other digital twins to make digital twins collaboration-based blockchain technology. For instance, we envision two possibilities for blockchain and digital twins. First, this requires information surrounding digital twins to be immutable (i.e., it cannot be modified); then, using blockchain plays a vital role in securing them. Therefore, instead of keeping it in a traditional database, one method is to save digital twin information in the blockchain, which can validate the data source. Furthermore, the importance of combining digital twins and blockchain is the need for digital twins to interact. Thus, the distributed ledger and smart contract are the two fundamental components of blockchain technology. As a result, smart contracts should be carried out in digital twins. Machine learning can also be used for predicting and detecting whether something happened in the digital twins.

The following five aspects highlight the benefits of combining digital twins, blockchain, and data analytics technologies:

(ii) *Data Security*. The decentralization feature of blockchain makes it harder for hackers to target critical data since hackers would need to compromise all digital twins, which is nearly impossible. Furthermore, any digital twin that behaves suspiciously is instantly expelled from the blockchain, which keeps the system in constant safety and security.
(ii) *Data Integrity*. Blockchain ensures data integrity by means of its encryption and stringent verification process. Further, it provides much-needed transparency through transaction traceability.
(iii) *Real-Time Data Analytics*. While blockchain offers real-time transactions, data analytics provides in-depth data analytics. These two technologies can be combined to deliver real-time data analytics that can revolutionize many industries and streamline business processes.
(iv) *Predictions*. Data analytics may be used to examine blockchain data to uncover essential data insights and hidden patterns.
(v) *Data Sharing*. Through storing data from data on a blockchain network, project teams may avoid reusing data that have already been utilized or duplicating data analytics that has already been done. In addition, the technique can assist in securely transmitting data without the requirement for repeated data cleaning.

With their benefits and drawbacks, blockchain and data analytics can be a strong combo for efficiently managing data quantity and quality. Furthermore, blockchain technology developments and maturity will enable the investigation of more use cases, including data analytics. Data analytics, on the other hand, can benefit blockchain because of its minimal storage costs. It will be fascinating to observe how these technologies develop in response to the present issues and demonstrate their ability to change data management and consumption.

### 2.3. Digital Twins Collaboration

**Figure 2:**
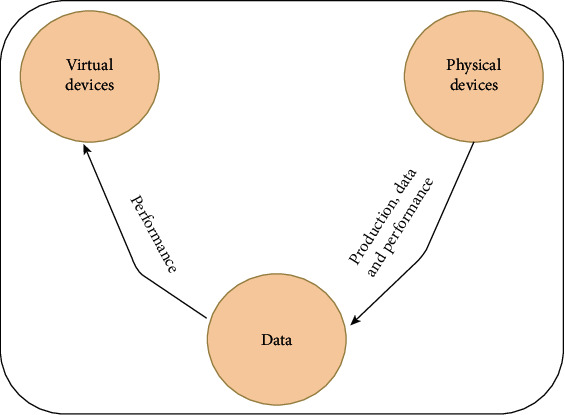
The main parts of digital twins [8].

Digital twins are a process of merging the virtual world and real world. It is used to describe the detailed presentation of machines, devices, robots in the warehouse, production, and process. The digital twins' advantages in Industry 4.0 include improving data security and quality, reducing cost, and faster decision-making. Schluse et al. [19] described digital twins as one virtual replica of a machines, robots, and devices containing data, function, and communication interfaces. The main parts of digital twins are a physical entity, virtual entity, and information that connect virtual and physical entities [8, 20, 21] as shown in Figure 2.

Collaboration means sharing and exchanging information among entities and sharing tasks to act accordingly. The collaboration of drones and the IoT is proposed to enhance the smartness of smart cities applications [22] and public safety and for better Quality of Service (QoS) [23]. The collaboration among multiuser and identification of the activities are described in [24]. Collaboration of digital twins and humans is described in detail in [25]. However, the authors highlighted that there are challenges of collaboration in industry platform [26–28].

Smart industries depend on data gathered from smart IoT devices or their digital twins of the production lines. The collected data can be erratic sensors, RFID, actuators, or their digital twins injecting and producing incorrect data for analyzing and making uneffective decisions. Based on this, Sahal et al. [8] has proposed a unified interaction mechanism for collaborative digital twins to provide an autodetection using the intelligence of operational data in the cyber-physical production system. Furthermore, the proposed mechanism can detect whether the digital twin has erratic behaviour through interacting with other collaborative digital twins within the edge level.

Catarci et al. [29] introduced architecture for smart factories, relying on service-based digital twins. The architecture presented how digital twins automatically combine the corresponding physical processes and share analogies with web services. Qi and Tao [30] introduced digital twin and big data in the smart industry, focusing on applications, manufacturing, production, maintenance prediction, and so on. Furthermore, Qi et al. [31] discussed the enabling technologies for digital twin in smart industries. He and Bai [32] also presented digital twin-driven sustainable smart industries. Moreover, Fuller et al. [33] introduced digital twins with Industry 4.0 and data analytics. However, none of the above studies addresses digital twins collaboration and applications. Therefore, we discuss digital twins collaboration based on blockchain for smart alerting systems.

## 3. The Proposed Framework of Blockchain-Based Collaborative Digital Twins for Pandemic Alerting

**Figure 3:**
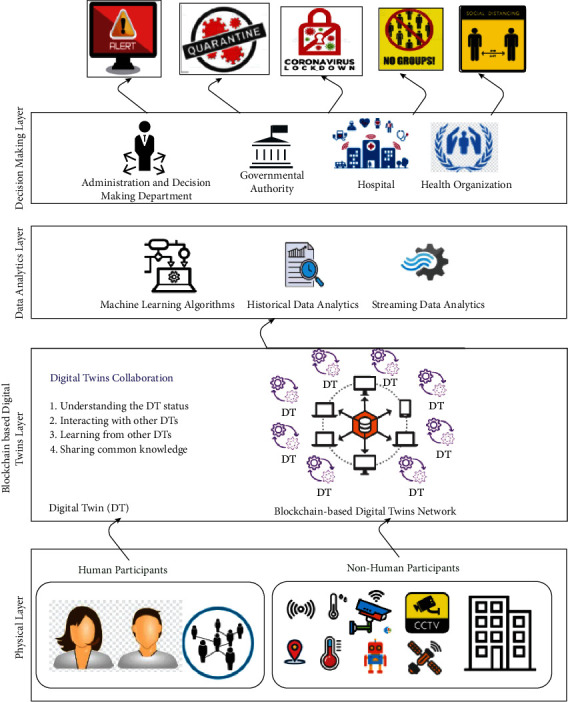
The architecture of the proposed conceptual framework in the medical cyber-physical system based on blockchain, digital twins collaboration, data analytics, and decentralized decision-making.

This paper proposes a conceptual framework of blockchain-based digital twins collaboration to enable alerting services for COVID-19-like pandemics and any future pandemics, such as announcing quarantine, lockdown, and restrictions. The proposed framework empowers more intelligent and collaborative solutions and decentralized decision-making. It introduces one higher level in the medical cyber-physical system to combat COVID-19 and any future pandemics. The merit of the proposed framework is exploiting the capabilities of blockchain to provide autonomous secure alerting service in the high level of secure, trusted, and transparent data exchange among the participants. The framework could be developed and implemented on top of any deployed blockchain and digital twins platforms. Four layers are used to equip the conceptual framework of blockchain-based digital twins collaboration with operational data intelligence. As shown in Figure 3, the four layers are the physical layer which contains human participants and nonhuman participants, the blockchain-based digital twins layer, data analytics layer, and decision-making layer. These layers will be elaborated as follows.

### 3.1. Physical Layer

The physical layer contains all nodes which are involved in the system. These nodes could be divided into human participants and nonhuman participants. The human participants include the people contributing using their operational data. For medical care system instance, the human participants could be people, patients, doctors, nurses, and employees in hospitals, health organizations, and the governmental sector. On the other hand, the nonhuman participants can be the monitoring devices (e.g., CCTV), sensor devices (e.g., temperature, humidity, and GPs), and robotic devices.

### 3.2. Blockchain-Based Digital Twins Layer

**Figure 4:**
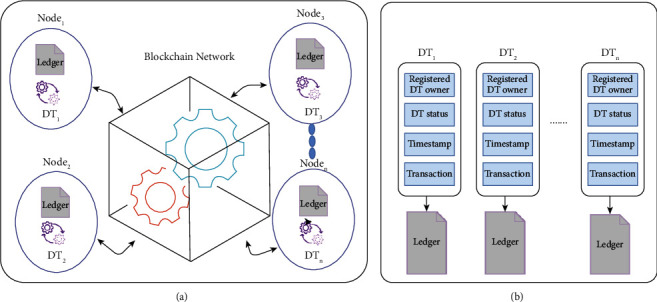
(a) Blockchain-based digital twins network and (b) ledger-based digital twins.

The blockchain-based digital twins layer contains a blockchain network for all connected digital twins, which represent the nodes (i.e., human participants and nonhuman participants) (see Figure 4(a)). In particular, multiple digital twins are connected through the blockchain network to maintain secure data exchange across multiple participants. According to Figure 4(b), each digital twin represents four attributes which are the registered digital twin owner, digital twin status, timestamp, and transaction [34]. The information that describes the physical node specification (e.g., digital twin owner) is stored in the ledger. Also, the transaction is stored in the ledger; it can be created by the communication between the digital twin and its physical counterpart, including the timestamped digital twins status. For digital twins collaboration, the digital twins collaboration concept within the proposed framework will help understand the digital twin status, interact with other digital twins, learn from other digital twins, and share common semantic knowledge within the medical, physical, and cyber systems.

Substantially, distributed ledger technology plays a vital role to guarantee secure and transparent communications between digital twins. The database within the ledgers is synchronously running to maintain the real-time updates among all digital twins. The updated status of the deployed digital twins contributes to the learning process to generate timely and accurate predictions sent to the decision-makers. For all digital twins from the same type or different types, the digital twins are defined in two aspects: data schema and collaboration. The data schema describes the attributes of the digital twin, which reflect the characteristics of its physical twin. The physical characteristics are divided into two types: manufacturing attributes which are static and operational attributes which are dynamic; they show the digital twin status (e.g., sensor readings). The digital twin collaboration scenario should be identified for the digital twin interaction with other digital twins (e.g., the communication activities).

### 3.3. Data Analytics Layer

**Figure 5:**
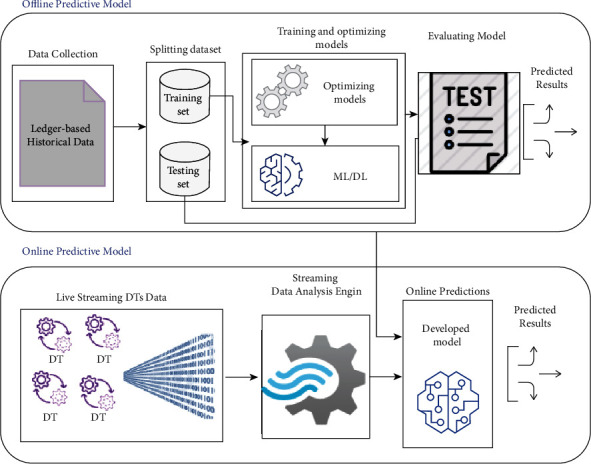
The workflow of building the predictive model using ledger-based data-driven digital twins collaboration.

This layer consists of a data-driven ledger-based predictive model to provide accurate predictions to the decision-makers. According to the digital twins collaboration context, the communication between digital twins at the edge level offers a history of communication activities stored with a ledger database and live streaming data to understand the digital twins' status [35]. In this layer, a ledger-based predictive model is demonstrated by developing two learning phases, the offline phase and online phase [36] (see Figure 5).

### 3.4. Offline Phase

For building an offline phase, a historical ledger database is utilized. A ledger is used to store the historical-based transnational data, which can be used for training models within the offline phase. A ledger database needs a query platform to efficiently retrieve stored transactions and manage limited data access like a traditional distributed database. Therefore, some research studies have been done to propose a ledger data query platform. For instance, a Ledgerdata Refiner, which is a ledger data query platform, was proposed to provide flexible query functions for real applications built on Fabric ledger [37]. According to Figure 5, the offline phase has four steps which are data collection, splitting dataset, training and optimizing models, and model evaluation.

(ii) *Data Collection*. A historical ledger database is utilized for training the models.
(ii) *Data Splitting*. The data are divided into training and testing sets.
(iii) *Training and Optimizing Models*. Machine learning techniques and deep learning techniques are used to predict potential risks, which helps in decision-making. The models are optimized using K-fold cross-validation and hyperparameter tuning techniques.
(iv) *Model Evaluation*. The model is evaluated using the standard machine learning metrics (i.e., accuracy, precision, recall, and F1-score).

Without loss of generality, the offline predictive model will be developed using distributed predictive model (i.e., classifier). And then, it will be trained using ledger-based historical digital twin operational data and evaluated in the smart contract.

### 3.5. Online Phase

On the other hand, the online phase is developed to evaluate the developed model using digital twin-driven live streaming data. One example of the predicted potential risk is the pandemic outbreak in a specific area. Based on the early prediction, the decision-makers can take proper actions such as the lockdown to combat the pandemic outbreaks.

### 3.6. Decision-Making Layer

The proposed framework introduces blockchain-based collaborative digital twins for pandemic alert, which provides a timely decision to combat pandemic outbreaks. Therefore, this layer is responsible for deciding if there is a severe pandemic outbreak. The participants within this layer can be governmental authorities, hospital management offices, health origination, administration, and decision-making departments. The decision is based on the potential risks of infected cases, which leads to announcements of alerts including quarantine, lockdown, or some restrictions to combat pandemic outbreaks. At the technical level, a distributed consensus algorithm is developed to improve the decision. In particular, the algorithm is developed based on the consensus principle [38]. Consequently, most participants' nodes have to agree on the alerting decision regarding the pandemic outbreaks. There are a set of consensus algorithms that could be used within this layer, such as Proof of Work (PoW), Proof of Stake (PoS), Practical Byzantine Fault Tolerance (PBFT), Proof of Elapsed Time, and Proof of Capacity.

## 4. Decentralized COVID-19 Pandemic Alerting Use Case

**Figure 6:**
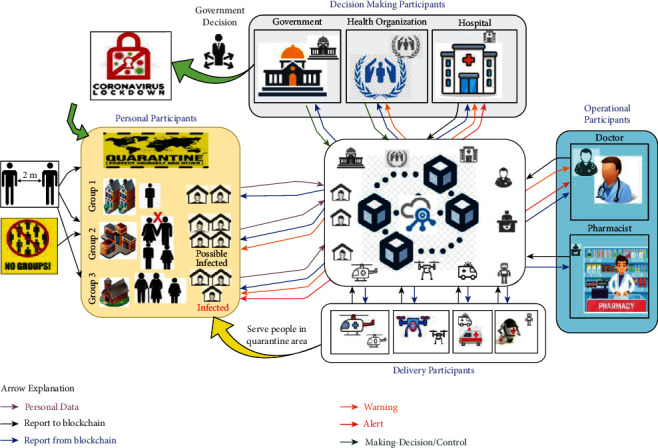
Decentralized COVID-19 pandemic alerting use case. Data are exchanged among the blockchain-based digital twins network. Arrow explanation: (a) purple arrow is for sending personal data, (b) black arrow is for sending report to the blockchain network, (c) blue arrow is for receiving the report from blockchain network, (d) orange arrow is for sending warning to the potential and infected cases and for sending warning of cases increase to doctors, hospitals, and health organizations, (e) red arrow is for sending alert to the infected case and for sending warning of cases increase to doctors, hospitals, and health organization, and (f) green arrow is for sending and broadcasting the decision (e.g., quarantine and lockdown) made by health organizations and governments to the blockchain network.

The proposed decentralized alert framework could be applied in critical situations like COVID-19 pandemic. Therefore, in this use case, we address the decentralized pandemic alerting to combat COVID-19. For this purpose, blockchain for digital twins collaboration can help produce pandemic alerts to notify the decision-makers in the governments. The decentralization feature is one of the advantages of blockchain technology to provide a high level of privacy and preserve transparency systems.  Figure 6 depicts the scenario of the decentralized pandemic alerting to combat COVID-19. Based on this, we will discuss a brief scenario of the decentralized epidemic alerting use case, taking COVID-19 pandemic as the actual use case. Before that, further details regarding mapping the decentralized COVID-19 pandemic alerting use case to the proposed framework are elaborated.

### 4.1. Mapping the Proposed Framework to the Decentralized COVID-19 Pandemic Alerting Use Case

This section presents a detailed use case scenario and the mapping of the proposed framework layers to the decentralized COVID-19 pandemic alerting use case.

#### 4.1.1. The Participants

**Figure 7:**
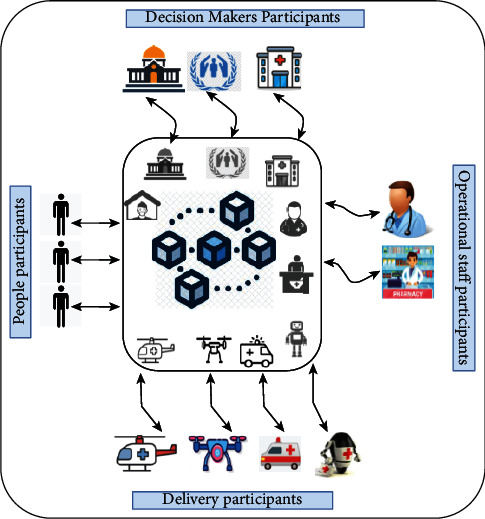
The participants of the decentralized COVID-19 pandemic alerting include decision-makers, people, operational staff, and delivery.

Based on the decentralized pandemic alerting to combat COVID-19, Figure 7 depicts the participants collaborating in the decentralized pandemic alerting based on the communication among their digital twins. These participants are decision-makers, people, operational staff, and delivery. They are represented in digital twins, sharing and exchanging their data using a blockchain network. Based on this, two main parts are required to equip the alert system, which are blockchain network and digital twins collaboration (see Figure 4(a)).

#### 4.1.2. Digital Twins Collaboration

Recently, Healthcare 4.0 refers to the use of Industry 4.0 technologies to improve healthcare systems. In particular, Healthcare 4.0 solutions aim to create interoperable and connected healthcare systems to make them more innovative. The processes within the healthcare systems generate big data collected by medical care tracking tools, including wearable devices and medical sensors. Based on Industry 4.0 principles, Healthcare 4.0 can be described as collaborative medical cyber-physical systems. Consequently, the digital twins of the participants are interacting and collaborating to perform the healthcare data, which is used for data-driven decisions.

Furthermore, the digital twins are used to monitor the status of the physical assets [10]. The physical assets are the participants including decision-makers, people, operational staff, and delivery in this use case. For instance, for the people participants, the digital twins provide the updated status of the people health conditions, such as their temperature.

#### 4.1.3. Data-Driven Digital Twins Model

According to work by Rasheed et al. [39], the digital twins could be modelled into four categories which are (1) physics-based modelling, (2) data-driven modelling, (3) big data cybernetics, infrastructure, and platforms, and (4) human-machine interface. In this work, three components are used to describe the data-driven digital twins model, which are digital model, data analytics, and knowledge base [40]. The data are generated from the sensors attached to participants (e.g., wearable devices), monitoring tools (e.g., CCTV), and machinery sensors. Based on the diversity of the participants, the model of a digital twin that represents a participant could be defined in a customized schema based on the attributes used to describe this participant.

#### 4.1.4. Blockchain-Based Digital Twins

Accessing patients' medical files is often a complex task due to critical data exchange across health data management parties [41]. Moreover, the healthcare data are fragmented into geographically distributed parties through the healthcare systems. For that, blockchain technology is used to allow the digital twins of the participants within the medical cyber-physical systems (e.g., people, doctors, healthcare providers, and decision-makers) to exchange and share their critical information quickly and safely. Blockchain technology can keep the information accessible, transparent, and secure among the network peers of the medical cyber-physical systems. According to the context of this work, the blockchain-based digital twin's network uses a collaboration-by-communication mechanism to securely exchange their data among the decentralized COVID-19 pandemic alerting participants.

#### 4.1.5. Predictive Data Analytics

Predictive data analytics in healthcare can help detect the potential risks to protect people's lives and avoid medical equipment downtime as happened during COVID-19 pandemic. According to the research work that conducted in healthcare-based predictive data analytics, it has been proven that predictive data analytics adds significant contributions to the medical decision-making process. For example, to assess the potential risks within the decentralized COVID-19 pandemic alerting use case (e.g., increase in the infected and potential cases), a prediction is needed to estimate COVID-19 symptoms and then decide whether the person has positive or negative COVID-19 test. Furthermore, based on the digital twin's collaboration context, the immutable historical data within the ledgers could train the machine learning models. In contrast, the collected live stream from the deployed digital twins is used to feed the online models to predict the potential infected cases and then inform the decision-making participants within the decentralized COVID-19 pandemic alerting use case.

#### 4.1.6. Consensus-Based Decision-Making

A consensus mechanism is a decision-making process performed by the participants to agree on a decision. It is used to keep the state of the ledgers synchronized among the blockchain network. Based on the decentralized COVID-19 pandemic alerting use case, the digital twins-based decision-making participants are responsible for providing the consensus-based decision (e.g., the announcement of strict lockdown of that area where the number of positive cases is highly increasing). In this work, we do not discuss the core ideas of the consensus algorithms. Rather, for brevity and simplicity, the consensus mechanism which could be used is the Practical Byzantine Fault Tolerance (PBFT) algorithm [42]. According to the PBFT algorithm, the potential risks (e.g., increase in the positive cases number) can be tolerated [43] and then the decision will be announced among the network peers within the decentralized COVID-19 pandemic alerting system.

### 4.2. Decentralized COVID-19 Pandemic Alerting Scenario

The participants in the decentralized COVID-19 pandemic alerting use case, including people (e.g., potential infected person, infected person, doctors, nurses, and pharmacists) and government organizations (e.g., hospitals and health organizations), communicate and share the updated status of COVID-19 to the blockchain network. Based on Figure 6, each digital twin of a participant sends its data to the blockchain network as depicted by the black arrows. The report from the blockchain network is sent back to the participant, as shown by the blue arrows. People in quarantine areas have permission to share their status to the blockchain networks using their predefined digital twins as it can be seen in the purple arrows in Figure 6. Doctors can access the status and suggest the required medicine to the smart contract. COVID-19 pandemic alert can also count the number of cases and identify the areas where COVID-19 is increasing.

In the pandemic alert, the analysis of updating status can be done on the data analytics layer. With the help of decentralization offered by blockchain technology and digital twins collaboration, the updated status of a person can be analyzed, and then the predicted result is sent to an epidemic alert if the informed case is detected. All of the people around will know the person who has a positive COVID-19 test and avoid touching and interaction. In particular, designing a machine learning model for analyzing data in a smart contract can be shared with all digital twins to investigate the case locally using edge computing. It depends on the used model to detect uninfected, potential COVID-19 infection, and confirm positive COVID-19 detection. Detecting cases by gathering local data can be done before the increase in outbreak. Therefore, the machine learning model can detect efficiently and actively through simultaneously monitoring people who may get sick around the confirmed positive cases [44]. Furthermore, the machine learning model can identify the location and track people around the detected case of positive COVID-19. Thus, virus infection origin can be tracked automatically, and people are notified of infected cases and potential via pandemic alert and digital twin.

Consequently, a couple of on-time remote alerts to notify all people around the infected or potential infected person can significantly limit COVID-19 outbreaks. According to Figure 6, both orange and red arrows denote the warning and alert, respectively. In case the digital twin shows that a person is infected with COVID-19, a couple of notifications will be distributed to everyone connected to the network. People around the infected COVID-19 person would be notified by their digital twin to start practising social distancing, stop touching things around, and spray disinfectant on their hands. Furthermore, digital twins of the people everywhere will analyze their status locally and send up-to-date information of the confirmed positive cases to the pandemic alert.

For example, based on Figure 6, Group 3 will receive a red warning alert due to a confirmed infected person who is one of the neighbours, where this alert warns the elder adults in specific because they are likely the age group at high risk. Group 2 will receive an orange warning alert to warn people to keep social distance. This warning alert is sent based on analyzing their movement and location, as their digital twins send their GPS and proximity sensors. Finally, Group 1 is not receiving any alert because there is no confirmed case around, and they are practising the social distance.

Receiving alerts in different places can control COVID-19 outbreaks. For instance, doctors can analyze the situation of detecting the digital twin of the infected person. The hospital can access the infected person digital twin data and prepare for receiving the infected person digital twin of COVID-19. Also, as it can be seen in Figure 6, hospitals and doctors can receive orange warnings and red alerts of COVID-19 increasing cases. Government and health organizations can get confirmation of the infected person from hospital reporting using his digital twin. Government can do the needful to keep COVID-19 pandemic under control such as the declaration of strict lockdown of the area where the number of positive cases is highly increasing. It can be seen in Figure 6 that the green arrow denotes the decision made by the decision-making participants, and then the decision is communicated to the blockchain network.

## 5. Related Work

In several industry areas, digital twin technology has been linked with blockchain technology [9]. ManuChain is a bilevel iterative methodology focused on incorporating blockchain into a digital twin on a decentralized manufacturing [45]. Using the decentralized properties of blockchain, the ManuChain model has established the lower-level for fine-grained self-organization intelligence and the upper-level to iterate the coarse-grained holistic optimization intelligence. Makerchain is yet another digital twin-based blockchain-based architecture that was suggested to handle the cyber-credit of social manufacturing among multiple makers [46]. To make manufacturing service transactions between makers more trustworthy, the Makerchain model has utilized digital twins to coordinate the updating of data tags and ensure customized requests.

Zhang et al. [47] suggested a manufacturing blockchain of things (MBCoT) architecture for safe, traceable, and decentralized manufacturing configuration. The data-and-knowledge-driven digital twin manufacturing cell has been defined as a reference model for decentralized manufacturing. MBCoT consensus-oriented transaction logic included the fault-tolerant protocol to help with autonomous production. Kuo [48] designed ExplorerChain, which contains online machine learning, transaction metadata, and the Proof-of-Information-Timed algorithm as a reference for academics interested in implementing and using blockchain technology in the healthcare/genomic sector. Obushnyi et al. [49] utilized DLT to create a protocol that ensures the transfer of values across digital twins in economic systems. Altun and Tavli [16] presented a ledger-based digital twin reference model for predictive maintenance that is divided into three layers: edge, fog, and cloud. Furthermore, a DLT-based architecture for safe digital twin data exchange is presented to monitor events and provenance information throughout an asset's lifespan, increasing transparency for all parties [5].

Using a library of component-based reduced-order models and interpretable machine learning, a predictive digital twins approach has been developed to allow data-driven physics-based digital twins [35]. However, during COVID-19, UAV plays a vital role in detecting absent masks, detecting the infected people of COVID-19, and monitoring people and social distance [7]. A smart city digital twin architecture is presented in [50] where knowledge representation, reasoning, and ML formalisms are utilized to provide complementing and supporting roles in data gathering and processing, detection, and automated decision-making. Digital twin cooperation has not yet been given much attention in the literature considering the modest number of papers in the blockchain-based digital twin. Furthermore, topics such as data interoperability-by-design have yet to be addressed.

Collaboration is required for a group of users to execute complicated tasks successfully and efficiently, which is something that a single user cannot achieve [24]. Therefore, Alsamhi and Lee [13] proposed blockchain technology for heterogeneous multirobot collaboration in decentralized peer-to-peer networks without human involvement to tackle COVID-19. In addition, Alsamhi et al. [7] used blockchain for decentralized multidrone collaboration to tackle COVID-19 in the delivery of products and surveillance of individuals in the quarantine zone. In addition, Alsamhi et al. [51] presented a machine learning approach for multirobot collaboration focused on preserving connection, maintaining service quality, and increasing mobility during job performing. In [7], the cooperation of drones and IoT devices was addressed to improve greener and smarter cities. Sahal et al. [34] have proposed a framework to empower more intelligent digital twins based on blockchain technology. They have described and validated their framework using smart transportation use cases in terms of smart logistics and railway predictive maintenance. However, none of the research mentioned above has dealt with blockchain-based digital twin collaboration and its applications for the pandemic alert. As a result, we discuss how digital twins may work together to improve decentralized COVID-19 pandemic altering systems.

Table 1 describes a comparison of the existing work and the present work concerning the applications, including blockchain, digital twins collaboration, and data analysis.

## 6. Discussion and Challenges

In this section, we discuss three issues regarding the challenges of the decentralized pandemic alerting framework.

### 6.1. Connectivity

With the proliferation of smart devices and digital twins, the connection remains a barrier for these devices to achieve their objectives in real time. Because the large number of smart devices and digital twins demands high connectivity, sophisticated communication technologies such as Beyond Fifth Generation (B5G) or Sixth Generation (6G) are required. If any smart devices and digital twins are disconnected, blockchain may assist them in borrowing data from nearby devices to keep the data transmission running smoothly. Machine learning at the edge might provide full connection and high accuracy and prevent data loss.

### 6.2. Timing, Speed, and Response

Timing and speed are difficult for the digital twins. For starters, time enhances decision-making and reaction times for customer service demands which require high accuracy and prompt replies.

### 6.3. Monitoring Technologies Capabilities

The monitoring technologies (e.g., sensors, CCTV, and drones) are considered the key of the data collections used to feed the learning system for the decision-making process. Monitoring technologies rely on the speed of data frequently capturing and the efficiency of communication among the network. However, some of the monitoring technologies can locally store the collected data for predefined time intervals. Therefore, a level of security risks may occur due to the collected information or the locally stored data. Consequently, efficient monitoring data technologies are used, better data-driven decisions are made, and timely alerts are sent.

## 7. Conclusion

In this work, we have proposed the conceptual framework of blockchain-based digital twins collaboration for pandemic alerting. The proposed framework utilizes the data-driven digital twins collaboration with the help of blockchain technology to combat pandemics such as COVID-19 outbreaks. Four layers are introduced to equip the conceptual framework of blockchain-based digital twins collaboration with operational data intelligence. The four layers are the physical layer which contains human participants and nonhuman participants, the blockchain-based digital twins layer, the data analytics layer, and the decision-making layer. Then, we describe how the conceptual framework can be applied in the decentralized COVID-19 pandemic alerting use case. In particular, we address the decentralized pandemic alerting to combat COVID-19.

## Figures and Tables

**Table 1:** Comparison of the existing work and our current proposed work.

Reference	Highlighted	Blockchain	Digital twin	Collaboration	Data analysis	Pandemic alerting
Leng et al. [45]	The ManuChain model is proposed based on the incorporation of blockchain into digital twin on a decentralized manufacturing	✓	✓	X	X	X
Leng et al. [46]	The Makerchain model based on digital twins is proposed to handle the cyber-credit of social manufacturing among various makers	✓	✓	X	X	X
Kuo [48]	Explorerchain is a reference for researchers to implement and deploy blockchain technology in the healthcare/genomic domain using online machine learning	✓	X	X	✓	X
Sahal et al. [8]	A framework for empowering intelligence digital twins collaboration based on blockchain for smart transportation	✓	✓	✓	✓	X
Zhang et al. [47]	MBCoT architecture for the configuration of a secure, traceable, and decentralized manufacturing	✓	X	X	X	X
Alsamhi and Lee [13]	A blockchain framework for heterogeneous multirobot collaboration to combat COVID-19 in decentralized peer-to-peer network	✓	X	✓	✓	X
Obushnyi et al. [49]	A protocol based on DLT to guarantee the transfer of values between digital twins in economic systems	✓	✓	X	X	X
Altun and Tavli [16]	A ledger-based digital twin reference model for predictive maintenance	✓	✓	X	✓	X
Sahal et al. [8]	A framework for automatic erratic operational data detection in Industry 4.0	X	✓	✓	X	X
Dietz et al. [5]	A framework for secure digital twin data sharing based on DLT to track events and provenance information	✓	✓	X	X	X
Kapteyn et al. [35]	A predictive digital twins methodology based on ML using UAV case study	X	✓	X	✓	X
Austin et al. [50]	A smart city digital twin architecture using reasoning and ML to automated decision-making	X	✓	X	✓	X
Our work	A framework of blockchain-based digital twins collaboration for smart decentralized pandemic altering	✓	✓	✓	✓	✓

## Data Availability

No data were used to support the study.
